# Solvent-Free Synthesis of Amidated Carboxymethyl Cellulose Derivatives: Effect on the Thermal Properties

**DOI:** 10.3390/polym11071227

**Published:** 2019-07-23

**Authors:** Asja Pettignano, Aurélia Charlot, Etienne Fleury

**Affiliations:** Université de Lyon, INSA LYON, Ingénierie des Matériaux Polymères IMP-UMR CNRS 5223, F 69621 Villeurbanne, France

**Keywords:** carboxymethyl cellulose, thermal amidation, plasticization, *T*_g_, polysaccharide modification

## Abstract

The present work explores the possibility of chemically modifying carboxymethyl cellulose (CMC), a widely diffused commercial cellulose ether, by grafting of hydrophobic moieties. Amidation of CMC, at high temperature and in heterogeneous conditions, was selected as synthetic tool for grafting on CMC a panel of commercially available amines (bearing long aliphatic chains, alkyl aromatic and heteroaromatic groups, more or less spaced from the cellulose backbone). The reaction was successfully carried out in absence of solvents, catalysts and coupling agents, providing a promising and more sustainable alternative to conventional amidation procedures. Relationships between the chemical structure of the obtained CMC derivatives and their thermal properties were carefully studied, with a particular attention to the thermal behavior. Grafting of aromatic and heteroaromatic alkyl amines, presenting a linear alkyl chain between CMC backbone and a terminal bulky moiety, allowed for efficiently separating the polysaccharide chains, improving their mobility and resulting in a consequent lowering of the glass transition temperature (*T*_g_). The *T*_g_ values obtained (90–147 °C) were found to be closely dependent on both the size of the aliphatic spacer, the structure of the aromatic ring and the extent of amidation.

## 1. Introduction

In the current context of sustainable development, the increased demand for greener alternatives to conventional materials and procedures is attracting a renovated interest towards the study of novel approaches for the design of bio-sourced products [1,2,3,4]. In this regard, cellulose, the most abundant polymer on earth [5], and its derivatives represent a promising source of raw materials, thanks to their high availability, low environmental impact, non-toxicity and presence of reactive groups. This last characteristic is particularly appealing, as their further chemical functionalization can be conveniently used to suitably modify their properties, potentially extending their applicability to new and unconventional uses [6,7]. Carboxymethyl cellulose (CMC), a cellulose ether in which some of the hydroxyl groups of the glucopyranose units are substituted by sodium carboxymethyl groups (–CH_2_COONa), is one of the most important cellulose ether derivatives, with 583.782 tons of annual global production and a wide range of applications (e.g., food industry, personal care, pharmaceuticals, textiles, paper, adhesives, ceramics) [8,9]. The presence of the carboxymethyl functions distributed along the polymer backbone confers to this derivative an increased reactivity and solubility in water, with respect to unmodified cellulose. Up to now, this reactivity has been mainly employed to modify NaCMC, by introduction of ester or amide functions, in homogeneous aqueous medium and in presence of carbodiimides as coupling agents [10]. Nevertheless, the lack of solubility of CMC in organic solvents, under both its sodium salt (NaCMC) and acid (HCMC) form, significantly limits its derivatization by grafting of hydrophobic molecules. Currently, only few strategies allow for the covalent grafting of hydrophobic moieties onto CMC, involving either the dispersion of the polysaccharide in organic solvent [11,12,13,14,15,16,17,18] or its previous modification (i.e., ion exchange to obtain the tetrabutylammonium (TBA) salt) [19,20,21] to form an organo-soluble derivative. Another possibility is represented by the condensation of CMC with an amine, at high temperature and in heterogeneous conditions, which results in the elimination of water and formation of an amide bond [22,23]. Despite the attractiveness of this strategy, which avoids the use of coupling agents, only few studies currently describe its employment for the functionalization of this cellulose derivative. Thermal amidation on CMC has been first reported in a patent of 1994 [24], where it was efficiently used for grafting mixtures of fatty amines (e.g., *n*-dodecylamine, *n*-hexadecylamine, oleylamine, *n*-octadecylamine, cocoamine, tallowamine, hydrogenated tallowamine, *N*-coco-1,3-diamino propane, *N*-tallow-1,3-diaminopropane, *N*-hydrogenated tallow-1,3-diamino propane, *N*-oleyl-1,3-diaminopropane). In the described protocol, an ammonium salt was first prepared, by putting in contact HCMC with the desired amine. The isolated salt was then transformed into the corresponding amide by heating it up to 140–150 °C, either after dispersing it in an organic solvent (i.e., ethylene glycol or *o*-xylene) or in solvent-free conditions. More recently, the thermal amidation of CMC was taken up by the groups of Sibikina [14] and Zabivalova [25], who both performed the amidation reaction without previous isolation of the ammonium salt, by simply dispersing HCMC in the solvent, in presence of the amine (i.e., aniline, benzylamine or *n*-decylamine), and subjecting the dispersion to the thermal treatment (i.e., dioxane at 100 °C or xylene at 140 °C). The study of the existing literature clearly indicates how the use of thermal amidation on CMC has been scarcely explored, with only few amine substrates having been tested and mainly in presence of organic solvents. Furthermore, to the best of our knowledge, none of the reported studies commented on the effect of the chemical modification on the properties of amidated CMC derivatives. Inspired by the potential interest that this challenging modification strategy could attract, the aim of the present study is to employ thermal amidation for preparing CMC derivatives, in a straightforward, solvent- and catalyst-free way. Wanting to address the versatility of this chemical functionalization route, a large set of amines with different chemical structures (i.e., aliphatic, alkyl aromatic and heteroaromatic) was tested and the thermal properties of the resulting derivatives were investigated for the first time. In particular, in order to evaluate the potential interest of the amidated CMC derivatives, a special attention was devoted to the effect of the chemical modification on their glass transition temperature (*T*_g_). More specifically, the impact of both the structural features of the grafted moieties and the extent of modification (quantified by the DS) on the *T*_g_ of functionalized CMC derivatives was studied in-depth.

## 2. Materials and Methods

### 2.1. Materials

Carboxymethyl cellulose sodium salt, having an *M*_w_ = 250 kDa and degree of substitution of 1.2, 0.9 or 0.7 (DS_CM_ = average number of hydroxyl groups substituted by carboxymethyl groups per one anhydroglucose unit), were supplied by Sigma Aldrich (St. Louis, MO, USA). Molecular sieves (4 Å, beads, 4–8 mesh), benzylamine, 2-phenylethylamine, 3-phenylpropylamine, 4-phenylbutylamine, undecylamine, dodecylamine, furfurylamine, 2-thiopheneethylamine were obtained from Sigma Aldrich. All the reagents were purchased at the highest commercial quality and used without further purification. Ethanol (96%), DMSO, ethyl acetate, toluene and HCl were supplied by Carlo Erba Reagents (Milan, Italy).

### 2.2. Acidification of NaCMC

Commercial NaCMC (1 g) was dispersed in a mixture of EtOH (80%, 100 mL) and HCl (37%, 10 mL) and stirred for 6 h [26]. The dispersion of HCMC was then poured in water (6 mL for each mL of EtOH/HCl mixture), in order to facilitate the recovery of HCMC by filtration. The recovered white powder was finally washed with acetone and dried overnight at 60 °C. 

### 2.3. General Procedure for Thermal Amidation Reaction on HCMC

HCMC (0.3 g, 1.6 mmol, 1 eq. –COOH), molecular sieves (4 Å, beads, 4–8 mesh, 1.4 g) and the desired amount of amine were placed in a 50 mL round bottom flask, allowing for the impregnation of the HCMC powder by the liquid amine for 15 min. When the employed amine was solid at RT, the mixture was slightly heated up above its melting point, to impregnate the powder. The flask was then transferred in a rotary evaporator and left to react, under rotating stirring (150 rpm) and at 140 °C, for 4 h. After cooling down to RT, DMSO (20 mL) was added to the flask to dissolve the modified polymer (slow magnetic stirring for minimum 6 h), which was then separated from the molecular sieves by filtration. The solution was poured in 300 mL ethyl acetate to precipitate the CMC amide, which was recovered by filtration and further washed in acetone (100 mL, replaced twice with fresh solvent, 5 h under stirring). The isolated powder was finally dried overnight at 60 °C. The experimental protocol described above was used to prepare all the amidated products. The products resulting from the amidation with undecylamine and dodecylamine were subjected to a different work-up procedure, consisting in washing the reaction mixture with toluene (20 mL, minimum 6 h) to remove the unreacted amine. After removing the molecular sieves with the aid of tweezers, the powder was washed with acetone (100 mL, replaced twice with fresh solvent, 5 h under stirring) and finally dried overnight at 60 °C. The mass yield of the amidated CMC derivatives was calculated by dividing the amount of product recovered after work-up by the attainable theoretical mass (calculated by taking into account the DS of the obtained product):

(1) $$\mathrm{Mass}\;\mathrm{yield}\;\left(\text{\%}\right)=\;\frac{\mathrm{Recovered}\;\mathrm{amount}\;\mathrm{of}\;\mathrm{product}}{\mathrm{Theoretical}\;\mathrm{amount}\;\mathrm{of}\;\mathrm{product}\;\left(\mathrm{considering}\;\mathrm{the}\;\mathrm{final}\;\mathrm{DS}\right)}*100$$

### 2.4. Characterizations

#### 2.4.1. Size Exclusion Chromatography (SEC)

The analysis was realized on a chromatograph equipped with two PL aquagel OH columns (mixed-M and mixed-H, 8 µm) and coupled with a combination of detectors (Light Scattering Instrument: HELEOS II and Refractive Index: Optilab rEX. Wyatt Technology Corporation, Santa Barbara, CA, USA). A 0.1 M NaNO_3_ buffer at pH 7 (filtered on a Cellulose Mixed Esters 0.1 µm syringe filter) was employed as eluent, with a flow rate of 0.5 mL/min. In order to verify that chain integrity is maintained during the thermal amidation reaction, a water-soluble control sample was obtained by subjecting commercial NaCMC to the same conditions employed for the reaction between HCMC and benzylamine (i.e., 0.3 g of NaCMC were soaked in benzylamine and stirred for 4 h at 140 °C, in presence of 4 Å MS). The powder was, then, manually separated from the MS, washed twice by stirring it in 150 mL acetone for 2 h each, and finally dried overnight at 60 °C. Solutions of native NaCMC and control sample were prepared in NaNO_3_ buffer at a concentration of 1 mg/mL, and filtered on a CME 0.45 µm syringe filter prior to analysis.

#### 2.4.2. Infrared Analysis (IR)

FT-IR spectra were recorded with a Nicolet iS10 (ThermoFisher Scientific, Waltham, MA, USA) by using attenuated total reflectance (Smart iTR Diamond), ATR, and collecting 32 scans from 600 to 4000 cm^−1^. 

#### 2.4.3. Nuclear Magnetic Resonance (NMR)

NMR spectra were recorded with a Bruker Advance 400 spectrometer (Billerica, MA, USA) (^1^H: 400 MHz, 358 K, 512 scans; ^13^C: 400 MHz, 358 K, 6144 scans; HSQC and HMBC: 400 MHz, 358 K, 32 scans) with DMSO-d6 as solvent. The chemical shifts (δ, ppm) are referenced to TSP-d4. DS values of the final products were calculated from ^1^H-NMR spectra as follow:

(2) $$\text{(i) }m_{\mathrm{graft}}=\frac{m_{\mathrm{TSP}-d4}}{M_{\mathrm{TSP}-d4}}x\frac{H_{\mathrm{TSP}-d4}}{I_{\mathrm{TSP}-d4}}x\frac{I_{\mathrm{graft}}}{H_{\mathrm{graft}}}{\mathrm{xM}}_{\mathrm{graft}}$$

(3) $$\text{(ii) }\mathrm{DS}=\;\frac{m_{\mathrm{graft}}/M_{\mathrm{graft}}}{(m_{\mathrm{total}}-m_{\mathrm{graft})}/M_{\mathrm{CMC}}}$$

where m values refer to the mass of the graft (as calculated by (i)), of the TSP-d4 and of the total polymer mass solubilized for NMR analysis. I_TSP-d4_ corresponds to the integral of TSP-d4 signal (which accounts for 9 protons), while I_graft_ is obtained by integrating one of the signals belonging to the graft (amide proton, for DS_Amide_, and a proton signal of the amine substituent, for DS_Sub_). H_TSP-d4_ and H_graft_ refer to the number of protons associated to each integrated signal. M represents the molar mass of TSP-d4, of the grafted amide and of CMC glucose unit, respectively.

#### 2.4.4. Thermogravimetric Analysis (TGA)

TGA measurements were performed on 10–15 mg of sample, using a TA Instruments TGA Q500 apparatus (New Castle, DE, USA) at a heating rate of 10 °C/min, up to 580 °C, under nitrogen (90 mL/min). The thermal decomposition temperatures Td_onset_ and Td_max_ stand for the temperature corresponding to the onset of the degradation and to the maximum to the derived curve, respectively. 

#### 2.4.5. Differential Scanning Calorimetry (DSC)

DSC analyses were carried out on a TA instrument Q10 or Q20 DSC (New Castle, DE, USA). The samples (3–5 mg) were placed in sealed aluminum cells. The amidated CMC derivatives were heated from 0 °C to 160–200 °C (two heating cycles), depending on the Td_onset_ values obtained by TGA for the different samples, and then cooled down to 0 °C (one cooling cycle) with a heating rate of 10 °C/min under nitrogen atmosphere. The glass transition temperature (*T*_g_) was determined from the second heating run and corresponds to the middle point.

#### 2.4.6. Dynamical Mechanical Analysis (DMA)

DMA experiments were performed with a DMA/SDTA861e Mettler Toledo (Columbus, OH, USA) apparatus, equipped for rectangular samples and working in shear mode. Values of the shear storage, G’, and loss moduli, G’’, as well as the tangent of the loss angle, tan δ = G”/G’, were determined. This apparatus was especially dedicated to the study of films and composite materials. Films of amidated CMC were prepared by solvent casting. Sample Benz_1.2_-CMC (Table 1, Entry 3) was dissolved in DMSO (10% *w*/*v*) by magnetic stirring for at least 18 h. The solution was poured in a silicone mold (2 cm × 2 cm) and left to evaporate at 80 °C for 48 h. Solvent evaporation in these conditions was slow enough to form a homogeneous and regular film. The obtained film was further heated up to 120 °C (temperature increased by 3 °C/min), under vacuum, maintaining the temperature for 5 h to ensure complete removal of DMSO and avoid any plasticizing effect due to the presence of the residual solvent. The films were finally cut and the average dimensions of the sample employed in DMA analysis are 7.2 × 6.8 × 0.1050 mm^3^. The tests were performed under isochronal conditions at 1 Hz and the sample was heated from room temperature to 180 °C at a heating rate of 3 °C/min.

## 3. Results

### 3.1. Thermal Amidation of Carboxymethyl Cellulose

**Figure 1:**
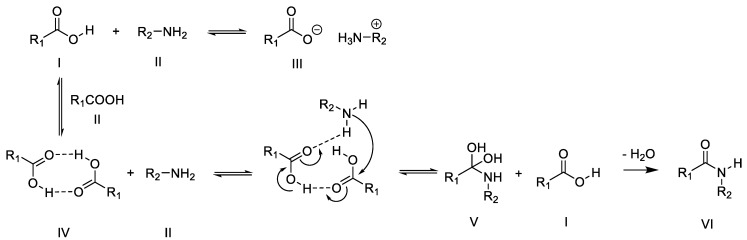
Mechanism of the thermal amidation of carboxylic acids.

**Scheme 1:**
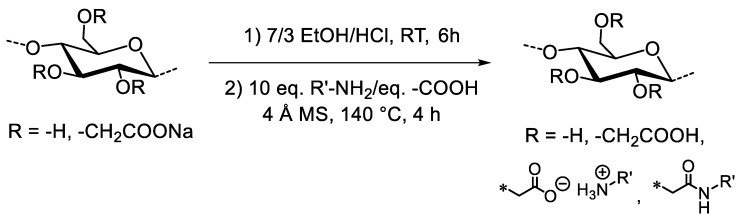
Experimental conditions used for thermal amidation of carboxymethyl cellulose (CMC).

Amidated CMC derivatives were prepared by the thermal treatment of a previously acidified CMC (HCMC) in the presence of a series of commercially available amines (Scheme 1 and Table 1, Entries 3–12), in absence of solvents, catalysts or coupling agents. The temperature-promoted modification of CMC is highly desirable, as it allows for the direct conversion of its carboxylic acid functions into the desired amides. Concerning the reaction mechanism (Figure 1), the most generally accepted starts with the carboxylic acid (I) and the amine (II), in equilibrium with the corresponding, unreactive, ammonium carboxylate salt (III) [22].

Carboxylic acid is activated by formation of a neutral, hydrogen-bonded dimer (IV), which undergoes nucleophilic attack by the amine (II). A concerted proton transfer from amine to acid and release of the second carboxylic acid leads to the formation of a neutral intermediate (V), from which water is readily lost to obtain the desired amide (VI). According to the reaction mechanism, water removal presents a crucial importance for the reaction outcome, as (i) it represents the driving force of the condensation reaction, shifting the reaction equilibria towards the desired product, and (ii) presence of water, even in small amounts, can cause the hydration of the H-bonded cyclic carboxylic acid dimer [22,27]. In order to achieve efficient direct amide formation, removal of water from the reaction can be performed either by azeotropic distillation [28] or by adding water scavengers, such as molecular sieves (MS), to the reaction mixture [22,29]. In the proposed protocol, the first step is represented by the acidification of commercial NaCMC (Table 1, Entry 1), to form the corresponding HCMC, performed by pouring the NaCMC powder in an ethanol/HCl mixture (See Section 2.2) and leaving it to acidify in heterogeneous conditions [26]. In contrast with NaCMC, HCMC is insoluble in water, which is employed as non-solvent to precipitate it as a white powder, with a mass yield of 86% (Table 1, Entry 2). The second step consisted in the amidation reaction, simply performed by soaking HCMC in the desired liquid amine, in presence of activated 4 Å MS, and by stirring at 140 °C, for 4 h. In order to ensure that the HCMC powder was homogeneously impregnated, a large excess of amine (i.e., 10 eq.) was used. Aiming to verify the influence of the structural features of the substituent on the reaction outcome and on the properties of the resulting products, the reaction was performed in the presence of a panel of commercially available amines, such as alkyl aromatic amines, characterized by different lengths of the alkyl spacer (1 to 4 -CH_2_- groups, Table 1, Entries 3–8), aliphatic amines (Table 1, Entries 9–10) and alkyl heteroaromatic amines (Table 1, Entries 11–12). Furthermore, NaCMC presenting different carboxymethylation degrees but equal molar mass (NaCMC, *M*_w_ = 250 kDa, DS_CM_ = 1.2, 0.9, 0.7, Table 1, Entries 5–7) were used, in order to elucidate the effect of DS_CM_ on the properties of a given amidated CMC derivative. At the end of the reaction, the obtained products were separated from the unreacted amine and the MS (as indicated in Section 2.3) and washed with acetone, to recover the desired products in moderate to high mass yields (Table 1, Entries 3–12, 57–76%). 

**Figure 2:**
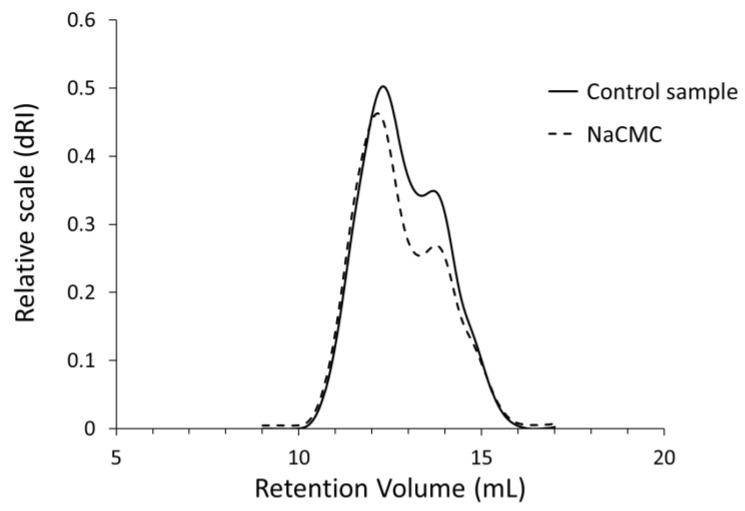
Size exclusion chromatography (SEC) curves of native NaCMC (-------) and NaCMC recovered after being left in contact with 13.5 eq. of benzylamine, for 4 h at 140 °C (―).

All of the obtained products, whatever the structure of the modifiers or the extent of modification, were found to be insoluble in water but soluble in polar aprotic solvents, such as DMSO and DMF. With the aim to verify the integrity of the polymer chain along the thermal amidation reaction, a water-soluble control sample was obtained by subjecting commercial NaCMC to the reaction conditions employed for the chemical modification (NaCMC soaked in benzylamine and stirred for 4 h at 140 °C in presence of 4 Å MS, See Section 2.4.1). The resulting CMC control was analyzed by size exclusion chromatography (SEC) in water and the chromatogram was compared with the one of unmodified NaCMC (Figure 2). The obtained SEC curves show the quasi-superimposition of the elution peaks of NaCMC and of the control sample, confirming the absence of any chain degradation phenomena in the used reaction conditions, which is a prerequisite for further chemical derivatization.

**Figure 3:**
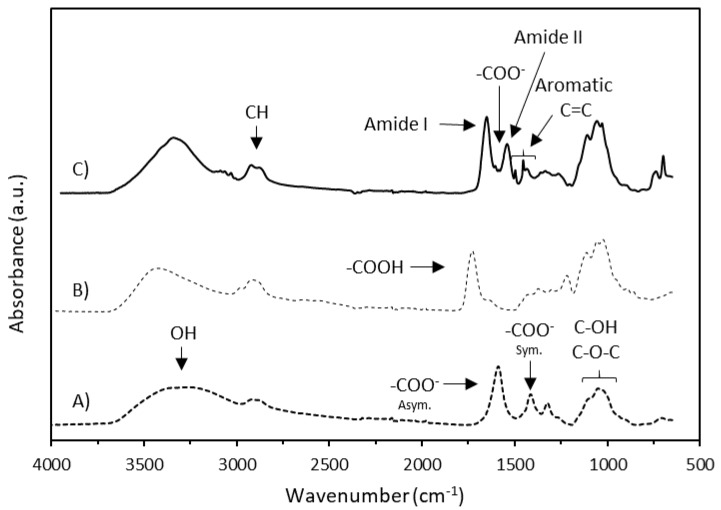
FT-IR of NaCMC (A,-------), HCMC (B,⋯⋯⋯) and CMC_1.2_-Benz (Table 1, Entry 3) (C,―).

Amidated CMC derivatives were firstly characterized by FT-IR and the product resulting from the reaction of NaCMC (DS_CM_ = 1.2) with benzylamine (CMC_1.2_-Benz, Table 1, Entry 3) was taken as an example to evidence the derivatization. The FT-IR spectra of NaCMC, HCMC and amidated product are shown in Figure 3. The comparison of the spectrum of HCMC with the one of pristine NaCMC shows the disappearance of the asymmetric –COO^−^ stretching band (at 1595 cm^−1^) and the presence of a new signal (at 1720 cm^−1^), corresponding to the C=O stretching vibration of the formed carboxylic acid groups, which confirms the success of the acidification step. The small shoulder at 1630 cm^−1^ is due to the presence of adsorbed water [30]. Regarding CMC_1.2_-Benz, C=C stretching vibrations of the aromatic ring can be observed between 1450–1500 cm^−1^ and the covalent coupling with the amine was proved by the appearance of two new intense bands at around 1660 and 1550 cm^−1^, attributed to the amide I and amide II signals, respectively, and by the concomitant disappearance of the carboxylic acid signal. Unreacted ammonium carboxylate salt could also be detected by the corresponding signal, at ≈1610 cm^−1^, overlapping the amide I and amide II bands. Nevertheless, in the CMC_1.2_-Benz sample, the unreacted ammonium carboxylate salt was present only in traces, indicating a high reactivity of the benzylamine substrate. The FT-IR spectra of the modified CMC derivatives (Figure 3 and Supplementary Material, Figures S1A–S7A) confirm the amidation reaction for all samples.

**Figure 4:**
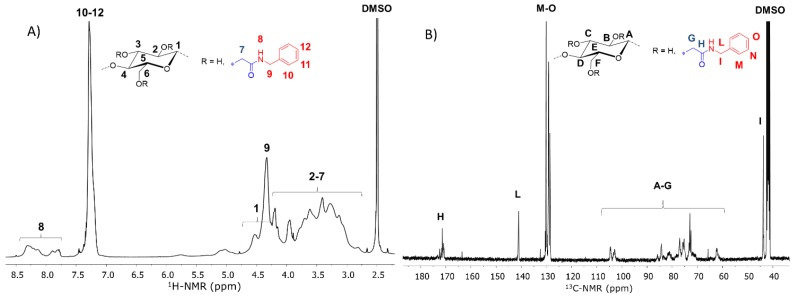
^1^H- (**A**) and ^13^C-NMR (**B**) of CMC-Benz (Table 1, Entry 3), in DMSO-d6 and at 358 K.

**Figure 5:**
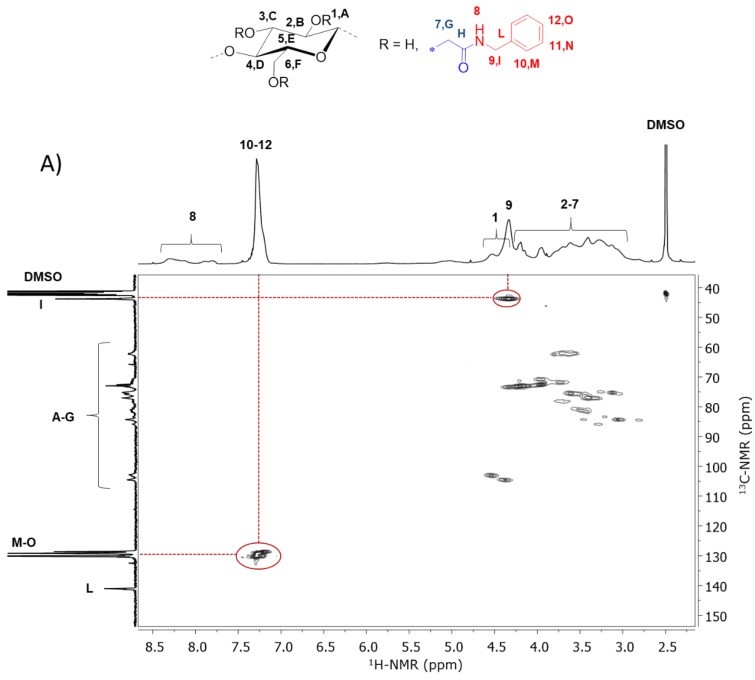

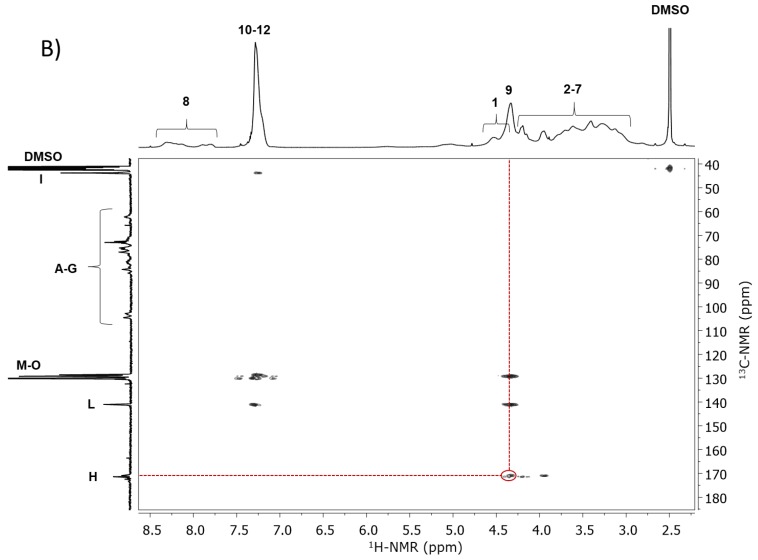
Heteronuclear Single-Quantum Correlation (HSQC) (**A**) and Heteronuclear Single-Quantum Correlation (HMBC) (**B**) spectra of product CMC-Benz (Table 1, Entry 3), in DMSO-d6 and at 358 K.

The structure of the modified CMCs was further analyzed by both one- (^1^H and ^13^C-NMR) and two-dimensional (HSQC and HMBC) NMR experiments, as shown in Figure 4 and Figure 5 for product CMC_1.2_-Benz, taken as an example. In the ^1^H-NMR spectrum (Figure 4A), the signals corresponding to CMC backbone are clearly visible between 2.7 and 4.8 ppm [31,32]. New signals assigned to the aliphatic (proton 9, at 4.3 ppm) and aromatic (protons 10–12, at 7.3 ppm) segments of the substituent could also be identified [33]. Furthermore, the appearance of signals attributed to the amide proton (8 between 7.7 and 8.4 ppm) unambiguously demonstrates the covalent bond between amine and the carboxylic acid functions of HCMC. ^1^H-NMR spectra of all the obtained CMC derivatives and the corresponding assignments are provided in Supplementary Material (Figures S1B–S7B). Similarly, the ^13^C-NMR analysis of CMC_1.2_-Benz (Figure 4B) shows the presence of the carbon signals of the expected benzyl group (carbons I at 44 ppm, L at 141 ppm, and M–O between 128–130 ppm) and of the carbonyl functions (carbon H at 170–172 ppm).

The analyses of ^1^H–^13^C heteronuclear single quantum coherence (HSQC), used to determine proton-carbon single bond (^1^J) correlations, and heteronuclear multiple bond correlation (HMBC), highlighting long-range (^2^J-^3^J) correlations between ^1^H and ^13^C nuclei, were employed to confirm the assignments of the substituent signals. HSQC analysis (Figure 5A) corroborates the chemical shifts of proton and carbon signals of the grafted moieties, by showing a direct correlation between protons 9 and 10–12 with the corresponding carbons I and M–O. The HMBC experiment (Figure 5B) not only further supports the assignments but allows for proving the covalent coupling through the multiple-bond correlation between benzyl CH_2_ (9) and the adjacent carbonyl of the amide (H). 

**Figure 6:**
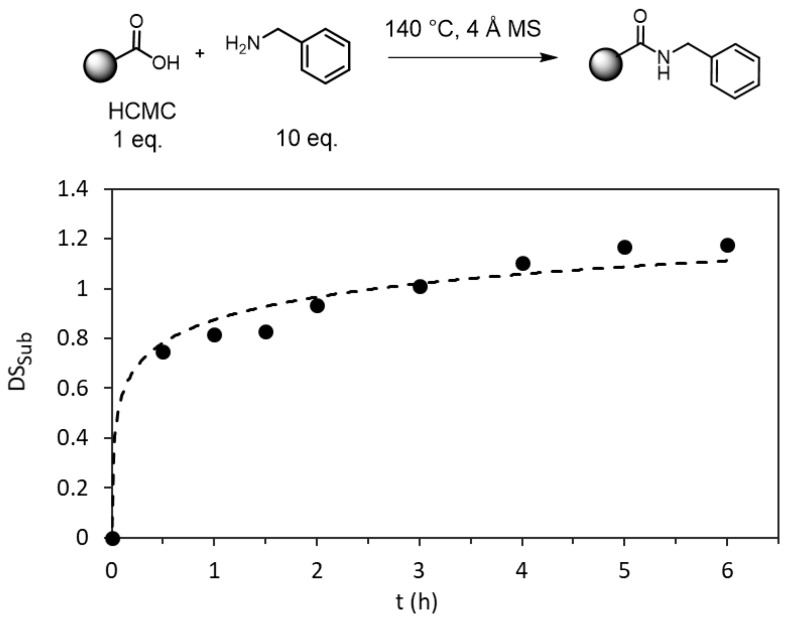
Evolution of DS_Sub_ with time for the reaction between HCMC and benzylamine. The dotted line serves as a guide to the eye.

^1^H-NMR spectroscopy was finally exploited to determine the degree of substitution of amidated products (DS_Amide_ and DS_Sub_), by using TSP-d4 as internal standard. The DS_Amide_ value was calculated from the integration of the amide proton (8) and of the signal belonging to the TSP-d4 reference, by taking into account the number of protons responsible for each integrated area (see Section 2.4.3 for more details on the calculation). Even employing anhydrous DMSO-d6 for the preparation of the NMR tubes, it must be pointed out that the DS value could be slightly underestimated, due to a certain H–D exchange of the amide proton in presence of eventual traces of water. Similarly, DS_Sub_ value has been calculated by integration of one of the signals corresponding to the substituent, with respect to the TSP-d4 signals. In this case, the calculation takes into account both the amine covalently bonded to CMC and, if present, the amine interacting with CMC under the form of ammonium carboxylate salt. In order to identify the optimal reaction time and investigate the kinetics of the process, the evolution of the DS_Sub_ was monitored over time (Figure 6). In the chosen experimental conditions, the reaction was found to rapidly proceed, reaching a DS_Sub_ of 0.75 after the first 30 min and a plateau (DS_Sub_ = 1.1–1.18) from 4 h of reaction.

The thermal amidation reaction was then extended to a series of different reagents, by varying the type of CMC (various degrees of carboxymethylation) and the structural features of the amine modifier (aliphatic, alkyl aromatic and heteroaromatic amines). Table 1 (and in particular the DS values) clearly shows that the developed synthetic route allows for the successful modification of CMC, leading to satisfying extents of modification and moderate to high substitution efficiencies, as a function of the used amine derivatives. In this regard, benzylamine was first tested by reacting it with HCMC samples, presenting different DS_CM_ (i.e., 0.7, 0.9, and 1.2). Whatever the initial DS_CM_, benzylamine is highly reactive, leading to substantial DS values and substitution efficiencies up to 85% (Table 1, Entries 3–5). For a given degree of CMC carboxymethylation degree (settled at 1.2), the impact of the length of the alkyl spacer was studied, by increasing it from C1 up to C2–C4 (Table 1, Entries 6–8). The employment of amines with a C2–C4 spacer generally resulted in a slightly lower reactivity (67–75% substitution efficiencies), with respect to benzylamine (83% substitution efficiencies). As shown in the reported FT-IR spectra (See Figure 3 and Supplementary Material, Figures S1A–S3A) the carboxyl functions of HCMC can either be involved in the formation of the amide bond (amide I and II signals, at ≈1660 and 1550 cm^−1^_,_ respectively), be transformed into the corresponding ammonium carboxylate salts (at ≈1610 cm^−1^) or remain as unmodified –COOH functions (1720 cm^−1^), depending on the reactivity of the amine. If in CMC_1.2_-Benz FT-IR spectrum the carboxylate salt results in a weak signal, the intensity increases when a longer spacer is used, indicating that larger amounts of salt are generated. This observation is also corroborated by the comparison between DS_Amide_ and DS_Sub_: if the two values are very close for CMC_1.2_-Benz (5% of difference), they differ significantly for longer-chain products (37–41% of difference), suggesting a larger abundance of the corresponding ammonium carboxylate salts and, therefore, their higher contribution to the calculated DS_Sub_ value. As previously reported in the literature [14,22], the basicity of the amine can heavily influence the rate of the direct amide formation, as the employment of amines with higher basic character could shift to the right the equilibrium (Figure 1) between the starting reagents (I and II) and the ammonium carboxylate (III), slowing down the formation of the desired amide. The higher reactivity of benzylamine in the amidation reaction, with respect to the other long-chain alkyl aromatic amines tested, can be thus probably found in its slightly lower basicity (pKa = 9.6 vs. pKa = 10.3–10.6) [34], leading to a lower stability of the corresponding ammonium carboxylates and, therefore, favored amide formation. Steric hindrance effects, induced by the long aliphatic chains bearing the bulky aromatic function, might also reasonably play a role in the observed lower reactivity, if compared with benzylamine. Two aliphatic amines, undecylamine and dodecylamine (Table 1, Entries 9–10), were then successfully ligated onto HCMC (DS_CM_ = 1.2), yielding a DS_Sub_ of 0.8 (67 % of substitution efficiency). The obtained DS_Sub_ value was supported by the FT-IR spectra (Supplementary Material, Figures S4A–S5A), showing the formation of a certain amount of ammonium carboxylate salt with both aliphatic amines. Taking into account the basicity of the two amines (pKa ≈ 10.6) [35], their behavior is consistent with what observed with alkyl aromatic amines, confirming the lower reactivity of long-chain amines in the reaction under study. The possibility of grafting two heteroaromatic amines, furfurylamine and 2-thiopheneethylamine (Table 1, Entries 11–12), was finally confirmed. Both amines could be coupled to HCMC with satisfying DS_Sub_ values (0.95 and 0.85, respectively, reflecting in an efficiency of 79% and 71%), coherent with those obtained for the analogous CMC_1.2_-Benz and CMC-PE samples. In summary, a library of amidated CMC-derivatives, with different chemical composition in terms of extents of modification and structural features, was successfully generated by a simple, one-step, non-degrading, solvent- and catalyst-free thermal amidation process.

### 3.2. Thermal Properties of Amidated CMC Derivatives

**Figure 7:**
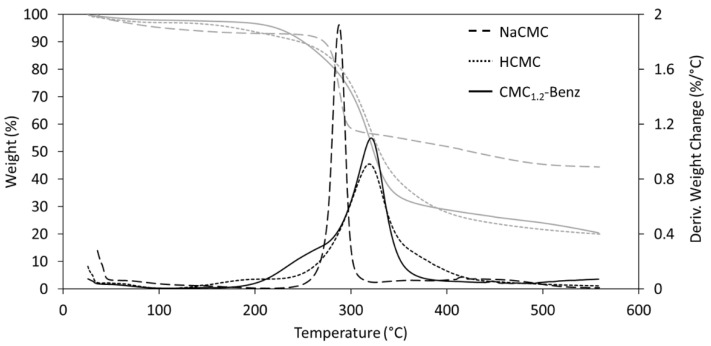
Thermogravimetric analysis (TGA) curves of NaCMC and CMC_1.2_-Benz (Table 1, Entry 3): Weight (%), grey lines, and Derivative Weight Change (%/°C), black lines.

Thermogravimetric analysis (TGA) under nitrogen atmosphere was exploited to evaluate the thermal stability of derivatized CMC derivatives and the corresponding thermal decomposition temperatures (Td_onset_ and Td_max_), as well as the decomposition temperatures of unmodified NaCMC and HCMC, are gathered in Table 1. Thermograms of NaCMC, HCMC and of an exemplificative amidated derivative (CMC_1.2_-Benz, Table 1, Entry 3) are displayed in Figure 7. As reported in the literature [36,37,38,39], the decomposition of NaCMC proceeds in two stages: (i) an initial weight loss, up to ≈190 °C (≈7%), attributed to the presence of adsorbed moisture and (ii) the decarboxylation of NaCMC and pyrolysis of the cellulosic backbone (up to ≈500 °C) [40]. On the other hand, the thermogravimetric analysis of HCMC evidenced the presence of a three-stage process: (i) desorption of adsorbed water, up to 100 °C, (ii) desorption of organic volatiles with low molecular weight, overlapping the (iii) degradation response of the acidified polysaccharide chain. With respect to NaCMC, HCMC is characterised by a wider degradation temperature range and higher Td_max_ (319 °C vs. 288 °C), possibly due to a stabilizing effect of the formation of intermolecular hydrogen bonds, induced by the presence of the carboxylic acid functions. Similarly, amidated CMC derivatives are characterised by a large thermal degradation profile, with a Td_max_ around 309–334 °C. With respect to pristine NaCMC and HCMC, amidated samples clearly show two intense overlapping signals, possibly indicating the initial degradation of the modified functions, either under the amide form or under ammonium carboxylate form, followed by the thermal degradation of the main carbohydrate chain. When compared to NaCMC, the amidated samples generally presented a higher Td_max_ (309–334 °C vs. 288 °C), regardless of the structure of the graft, as previously observed for HCMC. On the other hand, the amidated samples were all characterised by lower Td_onset_, with respect to NaCMC (225 °C). More in detail, aromatic compounds start to degrade around 180–190 °C, while aliphatic ones around 140–150 °C, coherently with the higher thermal stability of aromatic rings with respect to aliphatic chains [41]. This decrease in thermal stability can probably be attributed to the breakage of the interaction, covalent or ionic, between the substituent and the polysaccharide, with consequent loss of the bounded moieties. Despite the chemical grafting of hydrophobic moieties on CMC, the presence of a certain water uptake (from 1% to 5%) was detected for each sample, indicating that the modified CMC maintains a certain hydrophilic character. 

**Figure 8:**
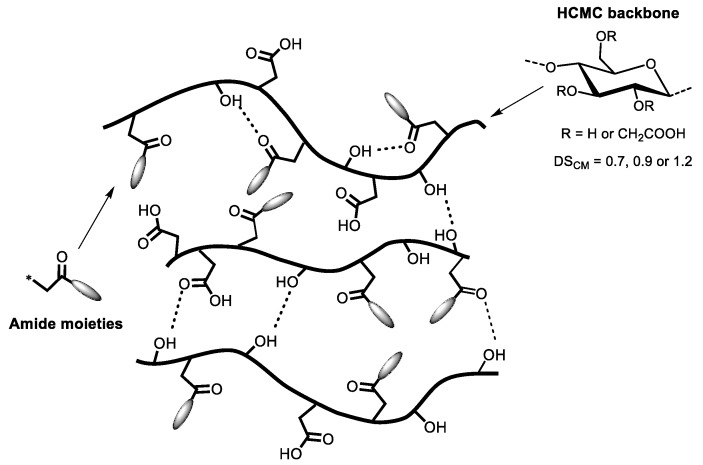
Schematic structure of internally plasticized CMC derivatives.

Although industrially available cellulosic derivatives (cellulose esters and, in particular, cellulose acetate) constitute an appealing bio-sourced alternative for the replacement of petroleum-based plastics, the presence of strong intermolecular and intramolecular hydrogen bonds is responsible of their high viscosity and elevated *T_g_*, requiring their previous plasticization to improve their processability as thermoplastics [42]. In this regard, besides the conventional use of plasticizing additives, grafting of molecules which act as “internal plasticizers”, by destroying the hydrogen bonds network and promoting the mobility of the macromolecular chains, has also been proved effective in lowering the *T_g_* of cellulosic derivatives [7,42,43]. For this reason, NaCMC, HCMC and the amidated CMC derivatives were analysed by differential scanning calorimetry, in order to assess the ability of the bound amine substituents to separate the polymer chains and increase their mobility (Figure 8).

**Figure 9:**
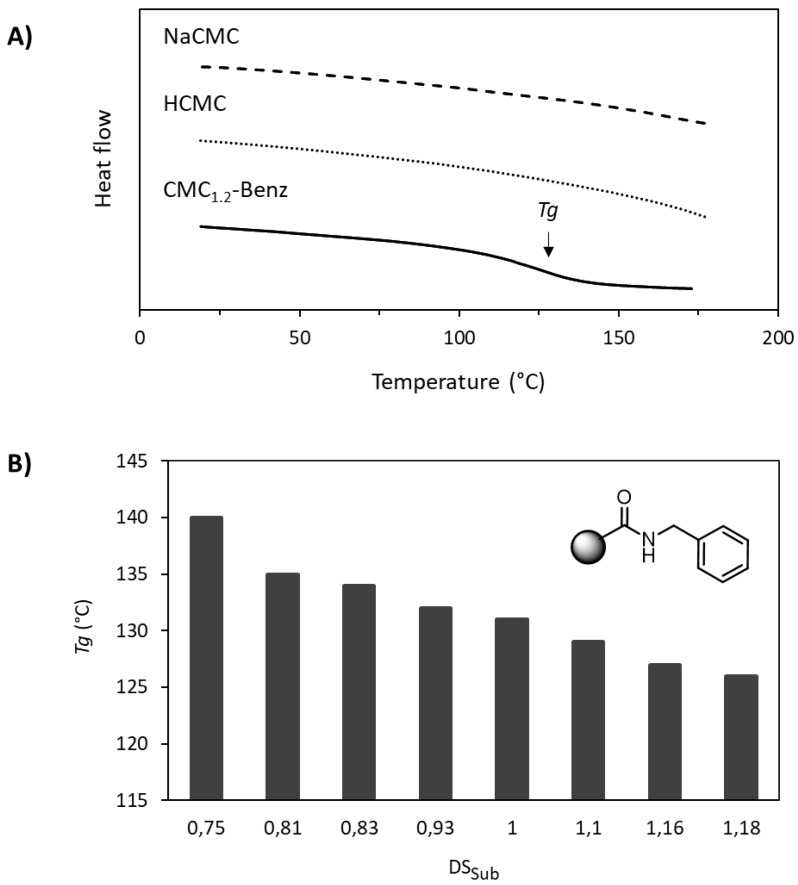
(**A**) Differential scanning calorimetry (DSC) curves of NaCMC, HCMC and CMC_1.2_-Benz (Table 1, Entry 3); (**B**) Evolution of *T*_g_ values of the amidated CMC-Benz derivatives as a function of DS_Sub_.

With the aim of avoiding any plasticizing effect due to the presence of adsorbed water, the glass temperatures values (*T*_g_) were determined as the middle point on the second heating ramp. The experimentally determined *T*_g_ values are summarized in Table 1 and some examples of DSC thermograms are given in Figure 9. Contrary to some previously published works that reported a *T*_g_ of 55–75 °C for NaCMC [38,44], our DSC analysis showed no glass transition on the second heating ramp cycle, for both NaCMC and HCMC (Figure 9A). However, as shown by the DSC thermogram of NaCMC (See Supplementary Materials, Figure S8), a slight transition can be observed on the first heating ramp (≈40 °C), followed by a broad endothermic band, attributed to the evaporation of water [45,46]. The absence of these two thermal phenomena on the second heating ramp confirms the plasticizing effect of adsorbed water on CMC.

**Figure 10:**
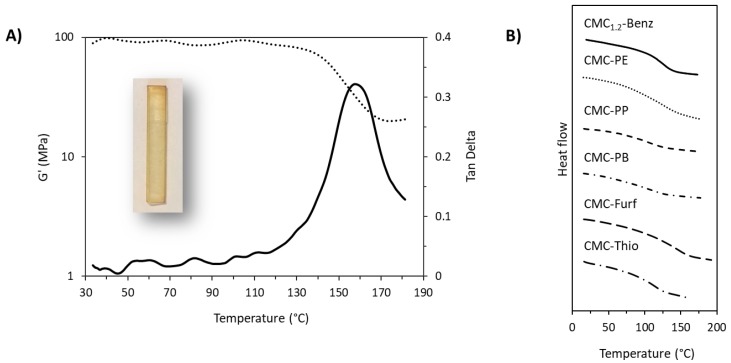
(**A**) Evolution of moduli G’ (- -) and of the loss tangent (tan δ) (―) as a function of temperature for a film made from CMC_1.2_-Benz (Table 1, Entry 3); (**B**) DSC curves of CMC_1.2_-Benz (Table 1, Entry 3), CMC-PE (Table 1, Entry 6), CMC-PP (Table 1, Entry 7), CMC-PB (Table 1, Entry 8), CMC-Furf (Table 1, Entry 11), CMC-Thio (Table 1, Entry 12).

As previously discussed, a decrease of *T*_g_ was expected after amidation, due to the introduction of side-groups which could possibly act as “internal plasticizers”. When long aliphatic chains amines were employed (C11–C12, Table 1, Entries 9–10) no *T_g_* could, surprisingly, be detected in the analysed temperature range (Supplementary Materials, Figure S9). This behaviour could possibly be ascribed to the formation of weak interactions, such as intermolecular interactions (e.g., Van der Waals interactions) between the side groups or hydrogen bonds between the amide functions and the hydroxyl groups of CMC backbone, which might outweigh the expected plasticizing effect. The attention was then focused on the impact of the structural features of the aromatic amines, more or less spaced from the CMC backbone. In this regard, the introduction of benzyl moieties (Table 1, Entry 3) induces a noticeable decrease in *T*_g_, which could be clearly detected around 129 °C (Figure 9A). Given the absence of residual molecules (i.e., solvent, residual water, unreacted amine), as confirmed by NMR and TGA analyses, such a decrease in *T*_g_ value can be exclusively ascribed to the effect of the bulky substituent, both in the form of amide or, if present, of ammonium carboxylate salt. Interestingly, the intensity of the plasticizing effect was found to be closely dependent on the extent of the chemical modification. When analyzing by DSC a series of CMC_1.2_-Benz samples, obtained at different reaction times (Figure 6), a progressive increase in DS_Sub_ (from 0.75 to 1.18) is accompanied by a concomitant decrease in *T*_g_ values (from 140 to 126 °C), as shown in Figure 9B. A similar effect can be underpinned by varying the DS_CM_ (i.e., 0.7, 0.9, and 1.2) of raw NaCMC employed for the reaction. The higher amount of available –COOH functions leads to higher grafting extent, that contributes to lower *T*_g_ *(*129 °C for DS_CM_ = 1.2 vs. 140 °C for DS_CM_ = 0.7) (Table 1, Entries 3-5). Although the study of the mechanical properties of the amidated CMC derivatives goes beyond the objectives of this work, a homogeneous free-standing CMC_1.2_-Benz (Table 1, Entry 3) film, prepared by solvent casting method from a solution in DMSO (Figure 10A; See Section 2.4.6), was characterized by dynamical mechanical analysis under shear mode. A controlled drying step was applied in order to ensure the complete removal of the solvent. For that, a DSC analysis of the dried film was achieved, confirming a *T*_g_ of ≈129 °C (See Supplementary Materials, Figure S10). The thermomechanical behavior of the film was monitored as a function of the temperature and the evolution of the storage modulus (G’) and tan δ are reported in Figure 10A. While no mechanical transitions could be evidenced on NaCMC films (See Supplementary Materials, Figure S11), the DMA thermogram of solvent-casted CMC_1.2_-Benz exhibits a single large α relaxation, characterized by a drop in G’ in the range between 135 and 180 °C, with a maximum of the tan δ around 160 °C. These values, linked to the glass transition region, are of the same order of magnitude than the *T*_g_ determined from DSC and indicate the increase in segmental mobility of the polymer chains. The significant width of the α relaxation can be associated to the mobility dispersion, possibly related to an uneven distribution of the substituents along the CMC chain.

If comparing the result obtained with benzylamine to the other alkyl aromatic amines, characterized by an increased length of the aliphatic spacer (from C1 to C4), we can observe how the structure of the substituent has a striking effect on the thermoplastic behavior of the modified CMC derivatives. The presence of a longer segment between the bulky moieties and CMC backbone induces a progressive decrease of *T*_g_, from 129 °C for a C1 spacer to 90 °C for the C4 one (Figure 10B), in spite of the lower DS_Sub_ values obtained (Table 1, Entries 3 and 6–8). Concerning heteroaromatic amines, the covalent anchoring of a furfuryl group (Table 1, Entry 11) is responsible of a moderate *T*_g_ decrease (147 °C, Figure 10B). Interestingly, a significantly low *T*_g_ (115 °C, Figure 10B) is obtained for CMC-Thio (Table 1, Entry 12), bearing a thiophene group, spaced from the CMC chain by a C2 alkyl segment. Previous studies demonstrated that the presence of bulky groups can play a dual role on the thermoplastic properties of cellulosic polymers: if the increase in the free-volume caused by the presence of bulky groups can lead to a decrease in *T*_g_, an elevated steric hindrance could limit their plasticizing effect [7,42]. The presence of a longer alkyl spacer between the bulky terminal moiety and the cellulose chain is therefore responsible of the higher flexibility and higher mobility of the modified product, that is reflected by a substantial lowering of *T*_g_. In consistence with the literature reports, we can conclude that derivatives consisting of a soft linear spacer between the main polymeric backbone and a terminal bulky aromatic group represent the optimal structure to simultaneously maximize free volume and mobility of the polymer chain.

## 4. Conclusions

Thermal amidation was shown to be a simple strategy for the chemical modification of CMC, allowing for the straightforward synthesis of a library of amidated CMC derivatives. A series of commercially available amines substrates, differing in their chemical structures (alkyl chains with different lengths, alkyl aromatic or alkyl heteroaromatic groups, more or less spaced from the CMC chain), was successfully introduced onto CMC, with short reaction times and in absence of solvents, catalysis and coupling agents. The efficiency of the amidation process was found to be dependent on the structure of the substituent, with amines presenting a long aliphatic chain showing a lower reactivity towards the desired amide. Among the different amines tested, benzylamine presented the highest reactivity, allowing for its efficient grafting on a series of HCMC substrates with different DS_CM_ (i.e., 0.7, 0.9, and 1.2). While the use of long chain aliphatic amines leads to CMC-amides with low thermal stability and no clearly detectable *T*_g_, the use of semi-aromatic amines allowed for the preparation of thermally stable CMC derivatives with thermoplastic behavior. Covalently bound bulky and rigid aromatic moieties, spaced from CMC backbone by an aliphatic segment, are able to increase the mobility of the polymeric chains, therefore reducing the *T*_g_ of the modified derivatives. The plasticizing effect was found to be highly dependent on both the extent of modification and on the structural features of the modifiers, with higher DS and longer aliphatic spacers contributing to lower the *T*_g_ values. In conclusion, the synthetic route described herein emerges as a promising technique for the solvent-free preparation of a wide range of CMC amides, with tailorable thermal properties.

## Figures and Tables

**Table 1:** Features of the amidated CMC derivatives.

Entry	Sample	R’	DS_CM_	Mass Yield (%)	DS_amide_^1^	DS_sub_^1^	Substitution Efficiency (%) ^2^	Td_onset_ (°C) ^3^	Td_max_ (°C) ^4^	*T_g_* (°C) ^5^
1	NaCMC	-	1.2	-	-	-	-	225	288	ND^6^
2	HCMC	-	1.2	86	-	-	-	ND^6^	319	ND^6^
3	CMC_1.2_-Benz	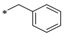	1.2	75	0.95	1	83	190	323	129
4	CMC_0.9_-Benz	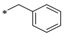	0.9	68	0.6	0.7	78	185	312	138
5	CMC_0.7_-Benz	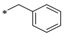	0.7	66	0.5	0.6	85	190	309	140
6	CMC-PE	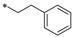	1.2	76	0.5	0.85	71	190	312	122
7	CMC-PP	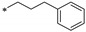	1.2	70	0.55	0.9	75	180	313	105
8	CMC-PB	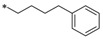	1.2	57	0.5	0.8	67	185	319	90
9	CMC-Und	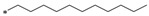	1.2	69	0.7	0.8	67	140	334	ND^6^
10	CMC-Dod	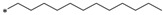	1.2	67	0.65	0.8	67	150	332	ND^6^
11	CMC-Furf	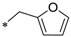	1.2	68	0.75	0.95	79	180	318	147
12	CMC-Thio	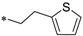	1.2	76	0.7	0.85	71	130	318	115

^1^ DS after thermal amidation, calculated by ^1^H-NMR spectroscopy, using TSP-d4 as internal standard (See Section 2.4.3); ^2^ Substitution efficiency (%) = DS_Sub_/DS_CM_; ^3^ Temperature corresponding to the onset of the degradation, determined by thermogravimetric analysis (TGA); ^4^ Temperature corresponding to the maximum of the derived TGA curve; ^5^ Glass transition temperature, determined by differential scanning calorimetry (DSC); ^6^ Not detectable.

## Supplementary Materials

The following are available online at https://www.mdpi.com/2073-4360/11/7/1227/s1, Figure S1: (A) FT-IR and (B) ^1^H-NMR (DMSO, 358 K, 512 scans) of CMC-PE, Figure S2: (A) FT-IR and (B) ^1^H-NMR (DMSO, 358 K, 512 scans) of CMC-PP, Figure S3: (A) FT-IR and (B) ^1^H-NMR (DMSO, 358 K, 512 scans) of CMC-PB, Figure S4: (A) FT-IR and (B) ^1^H-NMR (DMSO, 358 K, 512 scans) of CMC-Und, Figure S5: (A) FT-IR and (B) ^1^H-NMR (DMSO, 358 K, 512 scans) of CMC-Dod, Figure S6: (A) FT-IR and (B) ^1^H-NMR (DMSO, 358 K, 512 scans) of CMC-Furf, Figure S7: (A) FT-IR and (B) ^1^H-NMR (DMSO, 358 K, 512 scans) of CMC-Thio, Figure S8: DSC curves of the I and II heating scan of NaCMC, Figure S9: DSC curves of CMC-Und and CMC-Dod (II Heating Scan), Figure S10: DSC curve (II Heating Scan) of CMC_1.2_-Benz (Table 1, Entry 3) film employed for DMA analysis, Figure S11: Evolution of moduli E’ (- -) and of the loss tangent (tan δ) (―) as a function of temperature for a film made from NaCMC (Table 1, Entry 1).

## References

[1] Habibi Y., Lucia L.A. (2012). Polysaccharide Building Blocks: A Sustainable Approach to the Development of Renewable Materials.

[2] van den Broek L.A.M., Knoop R.J.I., Kappen F.H.J., Boeriu C.G. (2015). Chitosan films and blends for packaging material. Carbohydr. Polym..

[3] Kalia S., Avérous L. (2016). Biodegradable and Biobased Polymers for Environmental and Biomedical Applications.

[4] Elschner T., Obst F., Heinze T. (2018). Furfuryl- and Maleimido Polysaccharides: Synthetic Strategies Toward Functional Biomaterials. Macromol. Biosci..

[5] Klemm D., Schmauder H.-P., Heinze T., Vandamme E.J., De Baets S., Steinbüchel A. (2005). Cellulose. Biopolymers Online.

[6] Dhuiège B., Pecastaings G., Sèbe G. (2019). Sustainable Approach for the Direct Functionalization of Cellulose Nanocrystals Dispersed in Water by Transesterification of Vinyl Acetate. ACS Sustain. Chem. Eng..

[7] Boulven M., Quintard G., Cottaz A., Joly C., Charlot A., Fleury E. (2019). Homogeneous acylation of Cellulose diacetate: Towards bioplastics with tuneable thermal and water transport properties. Carbohydr. Polym..

[8] Heinze T., Liebert T. (2001). Unconventional methods in cellulose functionalization. Prog. Polym. Sci..

[9] Jadhav P. (2019). Global Carboxymethyl Cellulose Market 2019 by Manufacturers, Regions, Type and Application, Forecast to 2024.

[10] Pettignano A., Charlot A., Fleury E. (2019). Carboxyl-functionalized derivatives of carboxymethyl cellulose: Towards advanced biomedical applications. Polym. Rev..

[11] Charpentier D., Mocanu G., Carpov A., Chapelle S., Merle L., Muller G. (1997). New hydrophobically modified carboxymethylcellulose derivatives. Carbohydr. Polym..

[12] Merle L., Charpentier D., Mocanu G., Chapelle S. (1999). Comparison of the distribution pattern of associative carboxymethylcellulose derivatives. Eur. Polymer J..

[13] Rosilio V., Albrecht G., Baszkin A., Merle L. (2000). Surface properties of hydrophobically modified carboxymethylcellulose derivatives. Effect of salt and proteins. Colloids Surfaces B Biointerfaces.

[14] Sibikina O.V., Iozep A.A., Passet B.V. (2004). Reactions of Carboxymethyl Polysaccharides and Their Ethyl Esters with Amines. Russ. J. Appl. Chem..

[15] Ernsting M.J., Tang W.L., MacCallum N.W., Li S.D. (2012). Preclinical pharmacokinetic, biodistribution, and anti-cancer efficacy studies of a docetaxel-carboxymethylcellulose nanoparticle in mouse models. Biomaterials.

[16] Taubner T., Synytsya A., Čopíková J. (2015). Preparation of amidated derivatives of carboxymethylcellulose. Int. J. Biol. Macromol..

[17] Movagharnezhad N., Moghadam P.N. (2016). Folate-decorated carboxymethyl cellulose for controlled doxorubicin delivery. Colloid Polym. Sci..

[18] Leggio A., Belsito E.L., De Luca G., Di Gioia M.L., Leotta V., Romio E., Siciliano C., Liguori A. (2016). One-pot synthesis of amides from carboxylic acids activated using thionyl chloride. RSC Adv..

[19] Magnani A., Rappuoli R., Lamponi S., Barbucci R. (2000). Novel polysaccharide hydrogels: Characterization and properties. Polym. Adv. Technol..

[20] Barbucci R., Leone G., Vecchiullo A. (2004). Novel carboxymethylcellulose-based microporous hydrogels suitable for drug delivery. J. Biomater. Sci. Polym. Ed..

[21] Zhang C., Price L.M., Daly W.H. (2006). Synthesis and characterization of a trifunctional aminoamide cellulose derivative. Biomacromolecules.

[22] Charville H., Jackson D.A., Hodges G., Whiting A., Wilson M.R. (2011). The Uncatalyzed Direct Amide Formation Reaction - Mechanism Studies and the Key Role of Carboxylic Acid H-Bonding. Eur. J. Org. Chem..

[23] Allen C.L., Chhatwal A.R., Williams J.M.J. (2012). Direct amide formation from unactivated carboxylic acids and amines. Chem. Commun..

[24] Batelaan J.G., Van Der Horst P.M. (2000). Method of Making Amide Modified Carboxyl-Containing Polysaccharide and Fatty Amide Modified Polysaccharide So Obtainable.

[25] Zabivalova N.M., Bochek A.M., Kalyuzhnaya L.M., Vlasova E.N., Volchek B.Z. (2003). Carboxymethyl cellulose amides and their properties. Russ. J. Appl. Chem..

[26] Wu X., Wang M., Xie Y., Chen C., Li K., Yuan M., Zhao X., Hou Z. (2016). Carboxymethyl cellulose supported ionic liquid as a heterogeneous catalyst for the cycloaddition of CO2 to cyclic carbonate. Appl. Catal. A Gen..

[27] Chocholoušová J., Vacek J., Hobza P. (2003). Acetic Acid Dimer in the Gas Phase, Nonpolar Solvent, Microhydrated Environment, and Dilute and Concentrated Acetic Acid: Ab Initio Quantum Chemical and Molecular Dynamics Simulations. J. Phys. Chem. A.

[28] Arnold K., Batsanov A.S., Davies B., Whiting A. (2008). Synthesis, evaluation and application of novel bifunctional N,N-di-isopropylbenzylamineboronic acid catalysts for direct amide formation between carboxylic acids and amines. Green Chem..

[29] Cossy J., Pale-Grosdemange C. (1989). A convenient synthesis of amides from carboxylic acids and primary amines. Tetrahedron Lett..

[30] Heinze T., Liebert T., Koschella A. (2006). Esterification of Polysaccharides.

[31] Kono H., Oshima K., Hashimoto H., Shimizu Y., Tajima K. (2016). NMR characterization of sodium carboxymethyl cellulose: Substituent distribution and mole fraction of monomers in the polymer chains. Carbohydr. Polym..

[32] Kono H., Oshima K., Hashimoto H., Shimizu Y., Tajima K. (2016). NMR characterization of sodium carboxymethyl cellulose 2: Chemical shift assignment and conformation analysis of substituent groups. Carbohydr. Polym..

[33] Biallas P., Häring A.P., Kirsch S.F. (2017). Cleavage of 1,3-dicarbonyls through oxidative amidation. Org. Biomol. Chem..

[34] Wan H., Holmén A., Någård M., Lindberg W. (2002). Rapid screening of pKa values of pharmaceuticals by pressure-assisted capillary electrophoresis combined with short-end injection. J. Chromatogr. A.

[35] Matulis D., Bloomfield V.A. (2001). Thermodynamics of the hydrophobic effect. I. Coupling of aggregation and pK(a) shifts in solutions of aliphatic amines. Biophys. Chem..

[36] Biswal D.R., Singh R.P. (2004). Characterisation of carboxymethyl cellulose and polyacrylamide graft copolymer. Carbohydr. Polym..

[37] Uskoković V. (2008). Composites comprising cholesterol and carboxymethyl cellulose. Colloids Surf. B Biointerfaces.

[38] Su J., Huang Z., Yuan X., Wang X., Li M. (2010). Structure and properties of carboxymethyl cellulose/soy protein isolate blend edible films crosslinked by Maillard reactions. Carbohydr. Polym..

[39] Lin X., Li Y., Chen Z., Zhang C., Luo X., Du X., Huang Y. (2013). Synthesis, characterization and electrospinning of new thermoplastic carboxymethyl cellulose (TCMC). Chem. Eng. J..

[40] Ramiah M.V. (1970). Thermogravimetric and differential thermal analysis of cellulose, hemicellulose, and lignin. J. Appl. Polym. Sci..

[41] Moldoveanu S.C., David V. (2002). Chemical degradation of polymers and pyrolysis. J. Chromatogr. Libr..

[42] Chen Z., Zhang J., Xiao P., Tian W., Zhang J. (2018). Novel Thermoplastic Cellulose Esters Containing Bulky Moieties and Soft Segments. ACS Sustain. Chem. Eng..

[43] Vaca-Garcia C., Gozzelino G., Glasser W.G., Borredon M.E. (2003). Dynamic mechanical thermal analysis transitions of partially and fully substituted cellulose fatty esters. J. Polym. Sci. Part B Polym. Phys..

[44] El-sayed S., Mahmoud K.H., Fatah A.A., Hassen A. (2011). DSC, TGA and dielectric properties of carboxymethyl cellulose / polyvinyl alcohol blends. Phys. B Phys. Condens. Matter.

[45] Mura P., Gratteri P., Faucci M.T. (2002). Compatibility Studies of Multicomponent Tablet Formulations. DSC and experimental mixture design. J. Therm. Anal. Calorim..

[46] Siqueira E.J., Salon M.B., Mauret E. (2015). The effects of sodium chloride (NaCl) and residues of cellulosic fibres derived from sodium carboxymethylcellulose (NaCMC) synthesis on thermal and mechanical properties of CMC films. Ind. Crop. Prod..

