# Supplementary material for: Solvent-Free Synthesis of Amidated Carboxymethyl Cellulose Derivatives: Effect on the Thermal Properties
Source: Polymers (Basel). 2019 Jul 23;11(7):1227. doi: 10.3390/polym11071227 (PMC6680703; doi:10.3390/polym11071227)
Supplement: Supplementary file 1 [file polymers-11-01227-s001.pdf]

Supporting Information

Solvent-free synthesis of amidated carboxymethyl cellulose derivatives: effect on the thermal properties

Asja Pettignano, Aurélia Charlot, Etienne Fleury**

Université de Lyon, INSA LYON, Ingénierie des Matériaux Polymères IMP-UMR CNRS 5223 F 69621, Villeurbanne, France

*Corresponding authors: aurelia.charlot@insa-lyon.fr, etienne.fleury@insa-lyon.fr

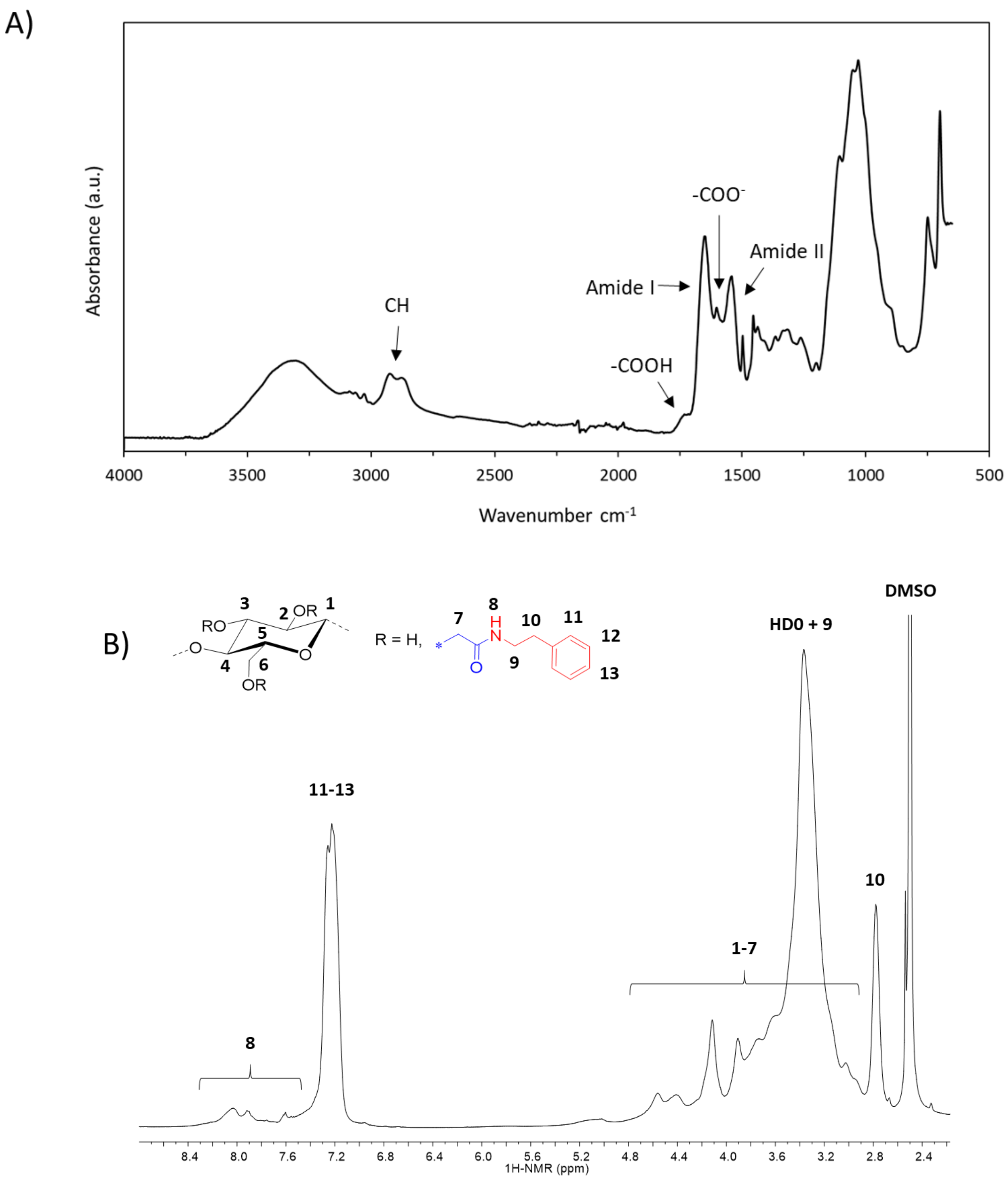

Figure S1: A) FT-IR and B) ^{1}H-NMR (DMSO-d6, 358 K, 512 scans) of CMC-PE

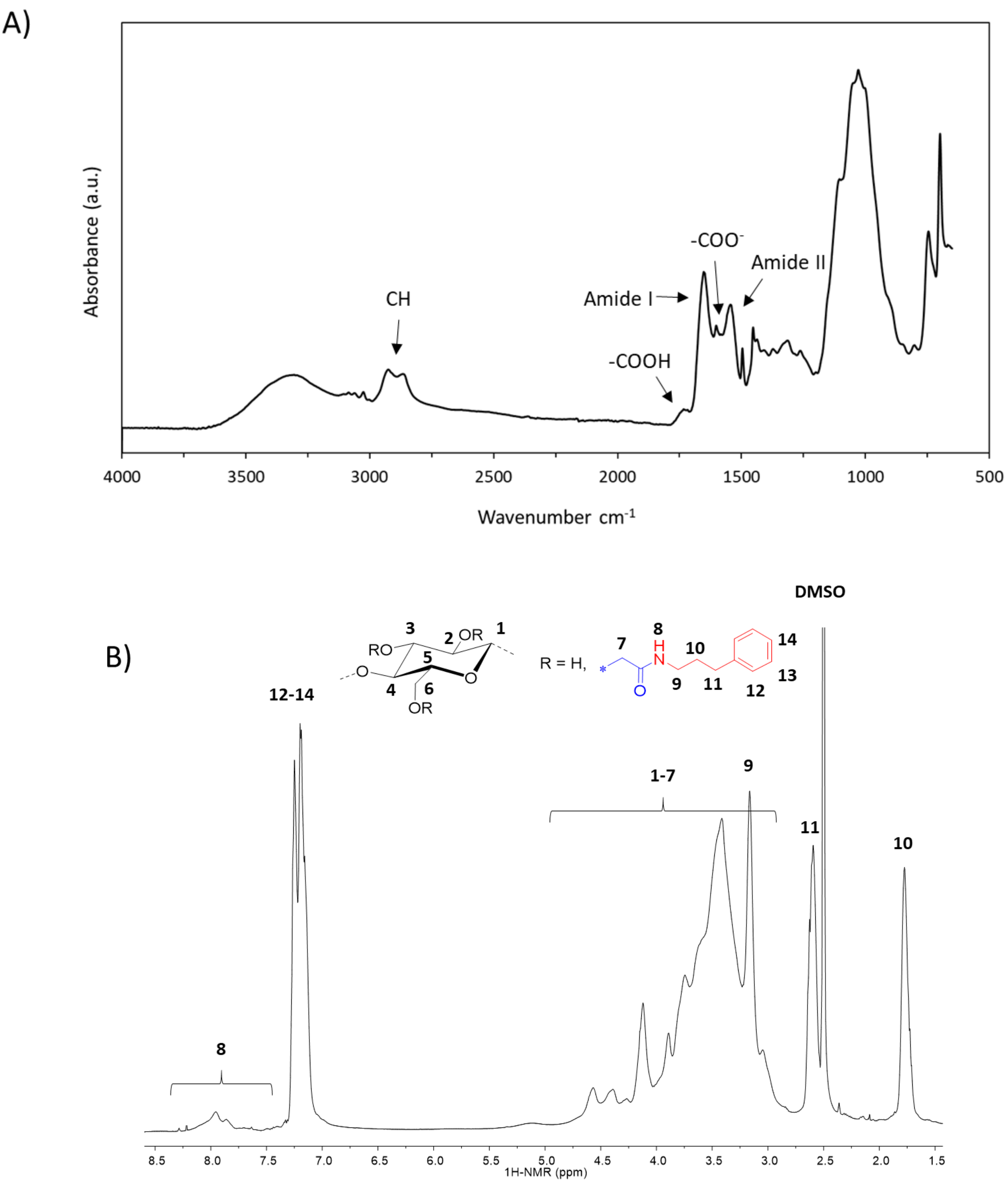

Figure S2: A) FT-IR and B) ^{1}H-NMR (DMSO-d6, 358 K, 512 scans) of CMC-PP

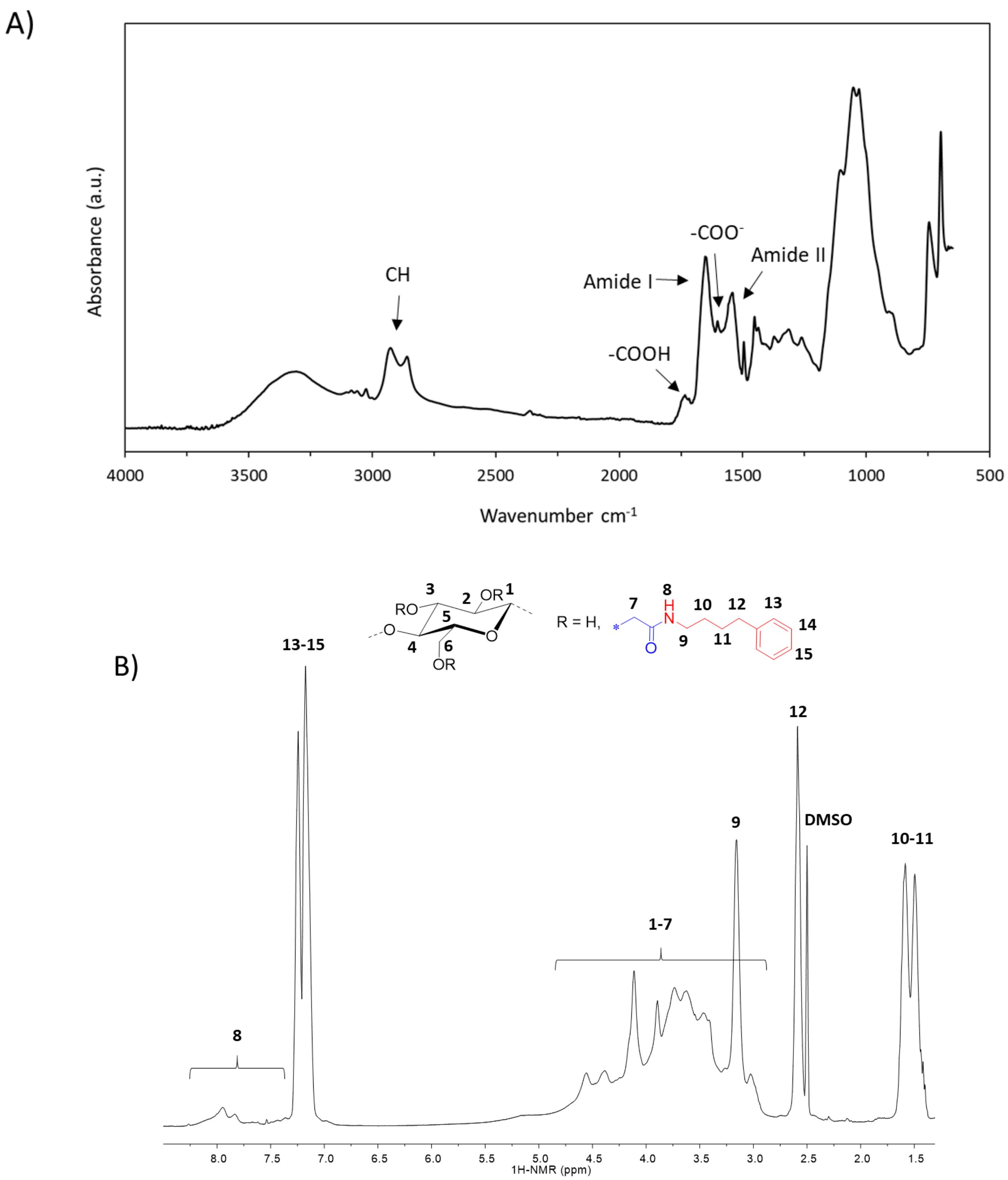

Figure S3: A) FT-IR and B) ^{1}H-NMR (DMSO-d6, 358 K, 512 scans) of CMC-PB

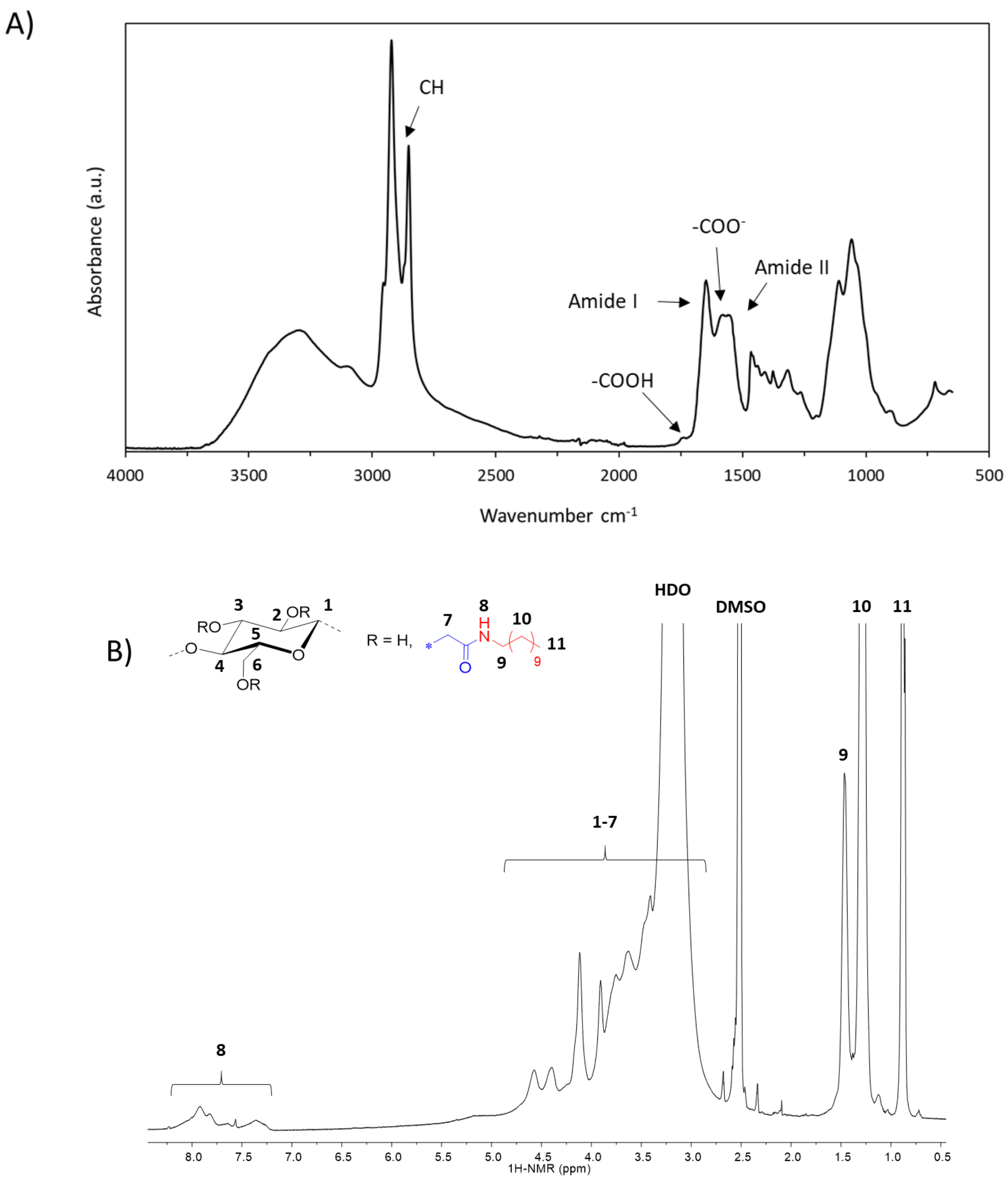

Figure S4: A) FT-IR and B) ^{1}H-NMR (DMSO-d6, 358 K, 512 scans) of CMC-Und

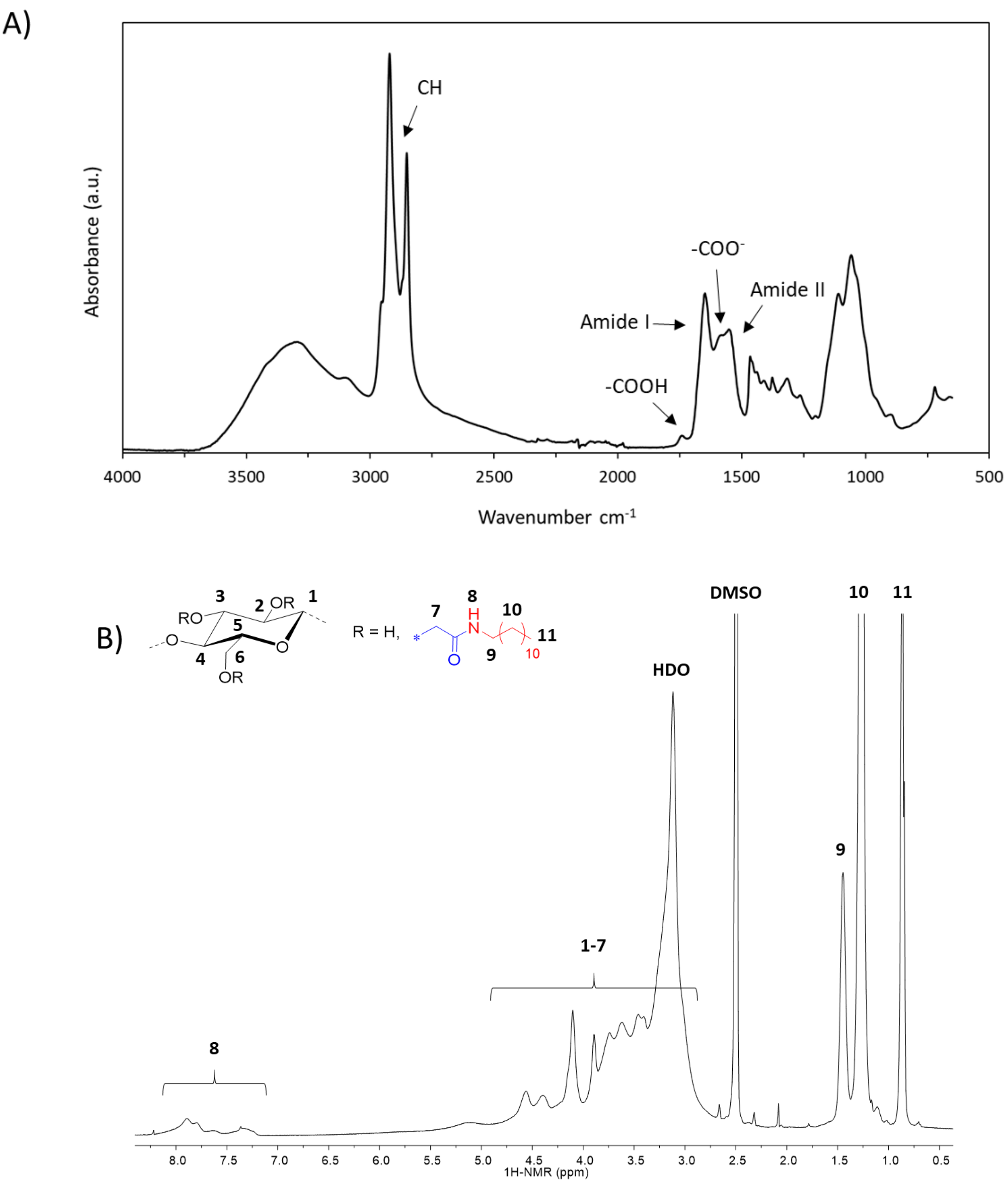

Figure S5: A) FT-IR and B) ^{1}H-NMR (DMSO-d6, 358 K, 512 scans) of CMC-Dod

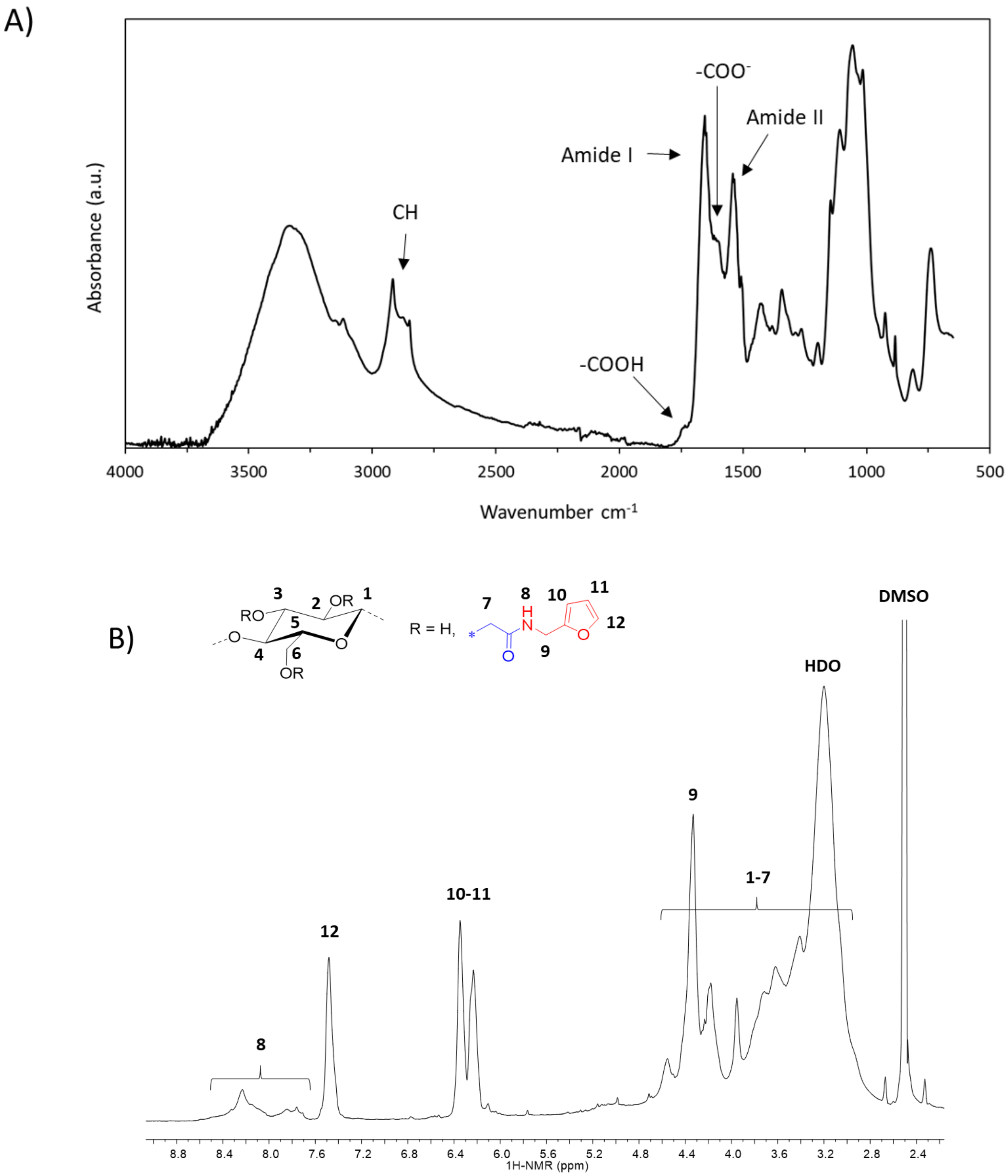

Figure S6: A) FT-IR and B) ^{1}H-NMR (DMSO-d6, 358 K, 512 scans) of CMC-Furf

A)

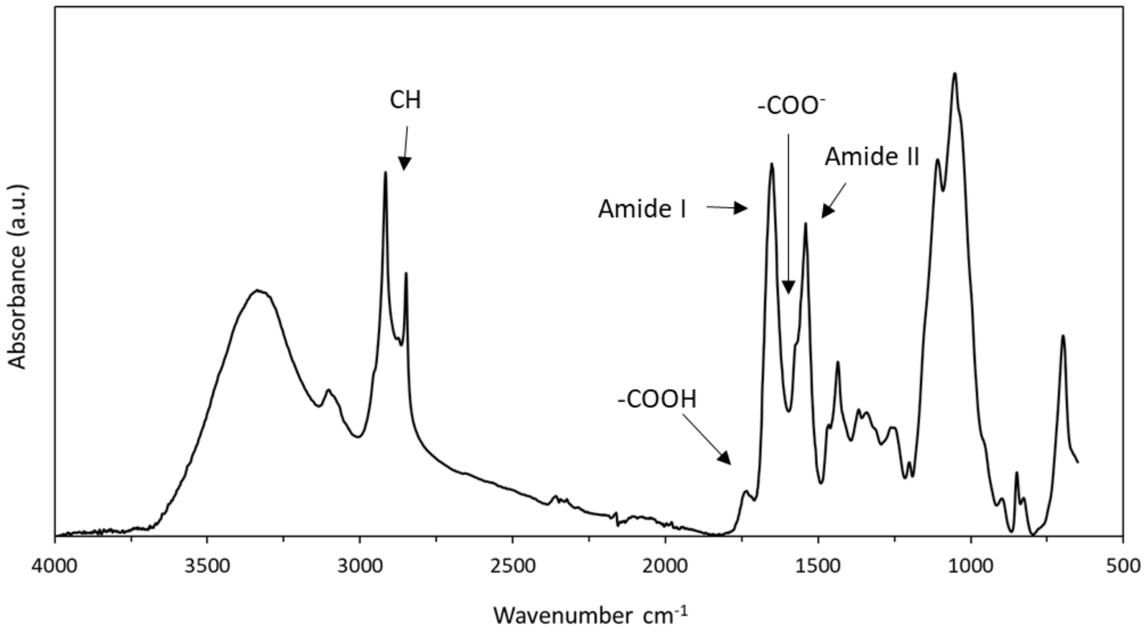

B)

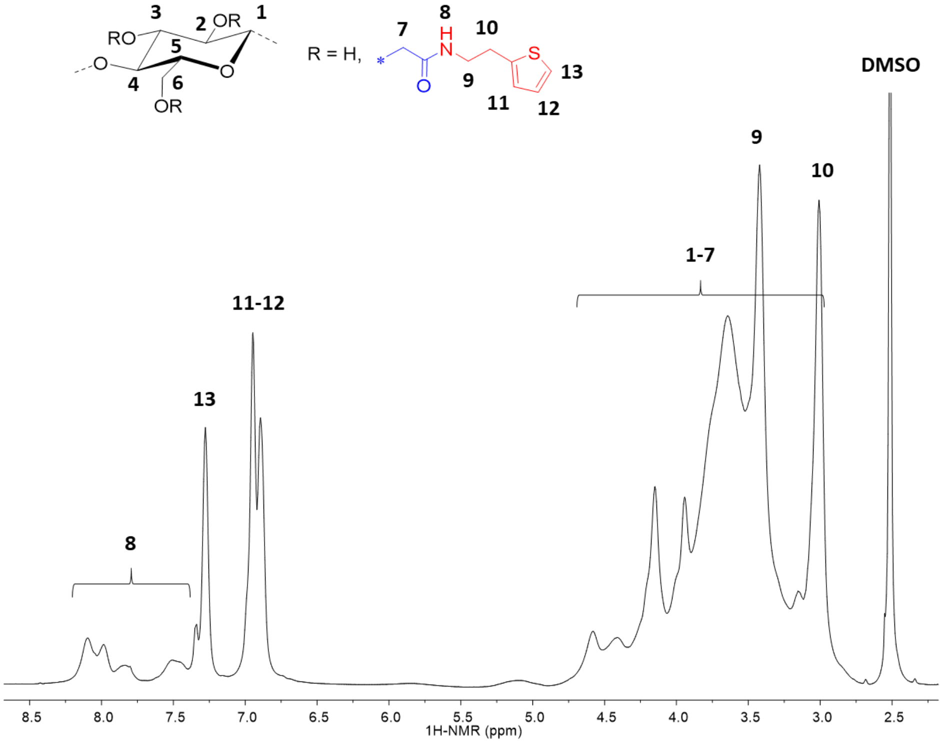

Figure S7: A) FT-IR and B) ^{1}H-NMR (DMSO-d6, 358 K, 512 scans) of CMC-Thio

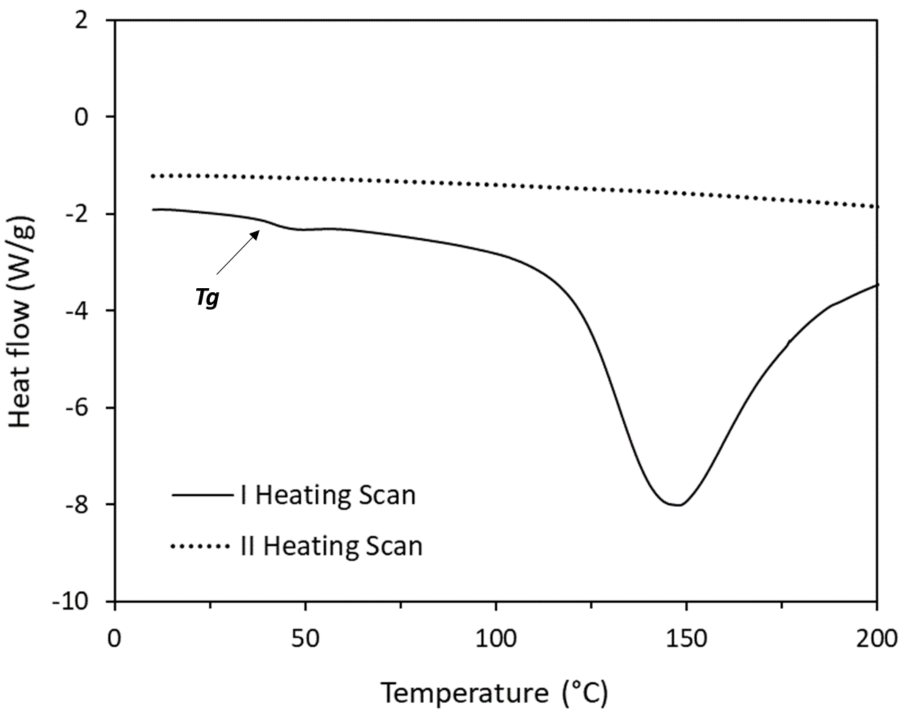

Figure S8: DSC curves of the I and II heating scan of NaCMC (Table 1, Entry 1)

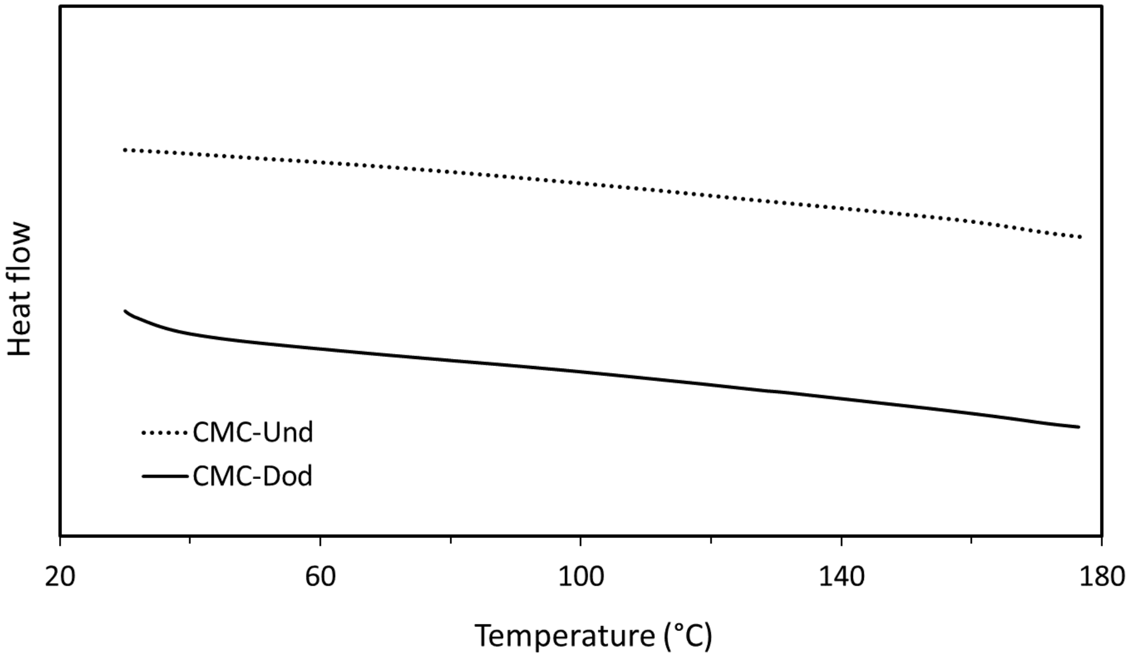

Figure S9: DSC curves of CMC-Und and CMC-Dod (Table 1, Entries 9-10; II Heating Scan)

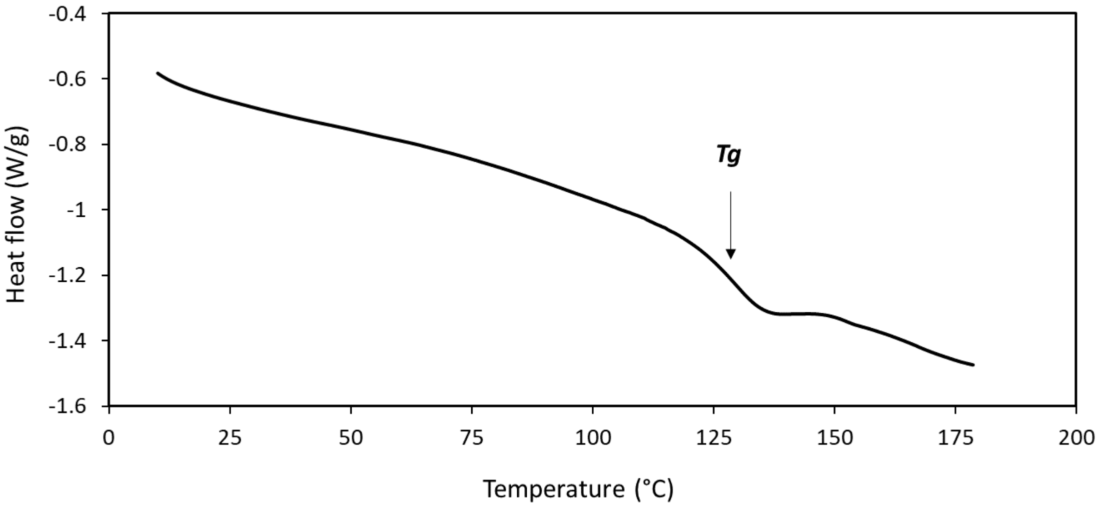

Figure S10: DSC curve (II Heating Scan) of $CMC_{1.2}$-Benz (Table 1, Entry 3) film employed for DMA analysis

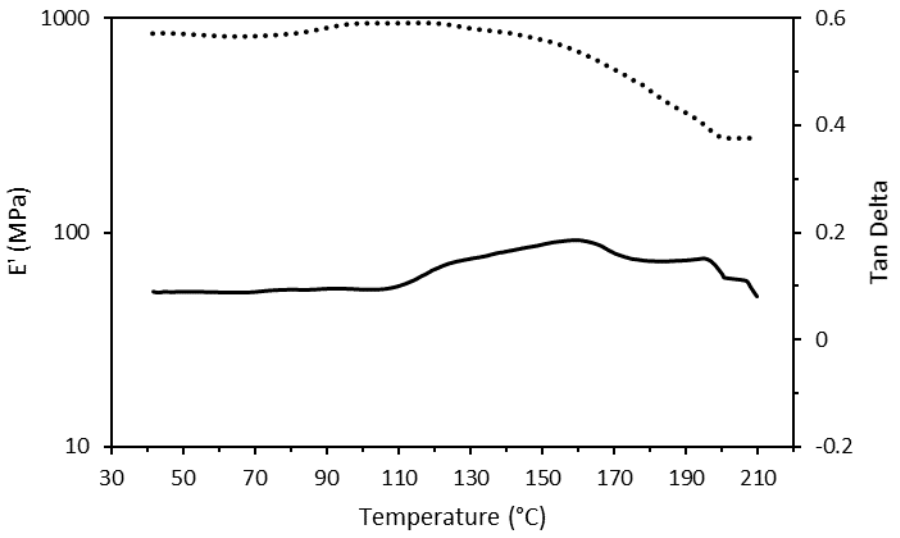

Figure S11: Evolution of moduli E' (- -) and of the loss tangent (tan δ) (—) as a function of temperature for a film made from NaCMC (Table 1, Entry 1). A DMA/SDTA861e Mettler Toledo apparatus, working in tensile mode, was employed for the analysis. Films were prepared by solvent casting, dissolving NaCMC in water (7 % w/v) and leaving it to evaporate in a silicone mold (2 cm x 2 cm), at RT for three days. Solvent evaporation in these conditions was slow enough to form a homogeneous and regular film. The tests were performed under isochronal conditions at 1 Hz and the sample was heated from room temperature to 210 °C at a heating rate of 3 °C.min^{-1}
